# Intravenously Administered, Retinoid Activating Nanoparticles Increase Lifespan and Reduce Neurodegeneration in the SOD1^G93A^ Mouse Model of ALS

**DOI:** 10.3389/fbioe.2020.00224

**Published:** 2020-03-27

**Authors:** David X. Medina, Eugene P. Chung, Collin D. Teague, Robert Bowser, Rachael W. Sirianni

**Affiliations:** ^1^Gregory W. Fulton ALS Center, Department of Neurobiology, Barrow Neurological Institute, Phoenix, AZ, United States; ^2^Barrow Brain Tumor Research Center, Barrow Neurological Institute, Phoenix, AZ, United States; ^3^Vivian L. Smith Department of Neurosurgery, University of Texas Health Science Center at Houston, Houston, TX, United States

**Keywords:** amyotrophic lateral sclerosis (ALS), retinoic acid signaling, nanoparticle, neurodegeneration and neuroprotection, drug delivery

## Abstract

Dysregulation of the retinoic acid (RA) signaling pathway is observed in amyotrophic lateral sclerosis (ALS) and other neurodegenerative disorders. Here, we investigated the therapeutic potential of retinoid activation via the RA receptor β (RARβ) in the SOD1^*G*93*A*^ mouse model of ALS. Our approach utilized the RARβ agonist adapalene, which we previously found to be neuroprotective *in vitro.* Adapalene, like most retinoids, is poorly water soluble, which has thus far prevented effective drug delivery *in vivo*. To address this challenge, we encapsulated adapalene within nanoparticles (Adap-NPs) composed of poly(lactic acid)-poly(ethylene glycol) (PLA-PEG). Our data demonstrate that intravenous administration of Adap-NPs robustly activates retinoid signaling in the CNS. Chronic administration of Adap-NPs resulted in improved motor performance, prolonged lifespan, and neuroprotection in SOD1^*G*93*A*^ mice. This study highlights retinoid signaling as a valuable therapeutic approach and presents a novel nanoparticle platform for the treatment of ALS.

## Introduction

Amyotrophic lateral sclerosis (ALS) is a fatal neurodegenerative disease for which effective interventions are lacking. Disease pathology results from the progressive loss of upper and lower motor neurons, which triggers degeneration of motor function and eventual death, typically 2–5 years following diagnosis (Kiernan et al., 2011; Pratt et al., 2012). The overall prevalence of ALS in the US is 5.0 per 100,000 and reaches levels of 20 per 100,000 in the 70–79 years age group (Mehta, 2018). While over 25 genes have been linked to familial or sporadic forms of ALS (Brown and Al-Chalabi, 2017), the molecular mechanisms involved in the pathogenesis of ALS remain poorly defined. Two drugs, Riluzole and Edaravone, are approved by the FDA for the treatment of ALS. However, these agents have limited clinical efficacy (Abe et al., 2017; Dharmadasa and Kiernan, 2018). There remains an urgent need to identify and engage new cellular targets that might curb the degenerative process.

The goal of this work was to test the hypothesis that activation of retinoid acid (RA) signaling in the central nervous system (CNS) would be neuroprotective in ALS. The RA pathway has been studied both in neurodevelopment and for its role in maintaining normal function of the adult nervous system (Maden, 2007). Established roles for RA signaling in the adult CNS include synaptic plasticity, learning and memory, neurogenesis and regeneration (Lane and Bailey, 2005). RA signaling has also been implicated in neurodegenerative disorders, including ALS. We previously demonstrated that nuclear localization of the retinoic acid receptor β (RARβ) is increased in motor neurons within post-mortem spinal cord tissue from ALS patients (Kolarcik and Bowser, 2012). RARβ nuclear localization occurred in motor neurons mostly devoid of markers for apoptosis, suggesting a neuroprotective role. To demonstrate RARβ’s neuroprotective role *in vitro*, we found that activation of retinoid signaling through the RARβ receptor significantly increased survival of primary motor neurons in response to oxidative stress. Together, these data suggest that that activation of retinoid signaling may be an adaptive, protective response to cell damage in ALS.

Here, we sought to develop the small molecule RARβ agonist adapalene for the treatment of ALS. Adapalene is FDA-approved for topical application in acne and cervical neoplasia. Adapalene is both poorly soluble and rapidly cleared from systemic circulation, which prevents effective systemic administration in free form (Ali et al., n.d.). To address these delivery problems, we engineered polymeric nanoparticles composed of poly(lactic acid)-poly(ethylene glycol) (PLA-PEG) and poly(lactic-co-glycolic acid) (PLGA) to encapsulate and slowly release adapalene (i.e., Adap-NPs). Our recent work confirms that intravenously administered, adapalene-loaded nanoparticles robustly activate retinoid signaling in the CNS (Medina et al., 2019). Here, we hypothesized that activation of retinoid signaling in the CNS via intravenous administration of Adap-NPs would be neuroprotective in a murine model of ALS. To test this hypothesis, we administered Adap-NPs to transgenic mice bearing a mutation in the superoxide dismutase gene (SOD1^*G*93*A*^ mice), evaluating treatment tolerability, motor performance, lifespan, and cellular responses to nanoparticle therapy, including histopathology and expression of neuroinflammatory markers. These data demonstrate that chronic treatment of SOD1^*G*93*A*^ mice with Adap-NPs improves motor performance, alleviates disease pathology, and prolongs lifespan. Intravenously administered Adap-NPs exhibited both neuroprotective and anti-neuroinflammatory action. Our work highlights retinoid signaling as a therapeutic target for ALS and emphasizes the important and potentially underutilized role of nanotechnology in drug development for neurodegenerative and neuromuscular disease.

## Materials and Methods

### Nanoparticle Fabrication

We utilized a polymer blending approach to generate PLGA/PLA-PEG nanoparticles with optimized properties via single emulsion-solvent evaporation. This technique was previously described (Medina et al., 2019). Briefly, 50 mg of polymer (PLA-PEG 16:5 kDa:PLGA 1–5 kDa blended in a 6:4 ratio) (PolySciTech) and 2 mg of adapalene were dissolved in dichloromethane. The solution of dissolved polymer with drug was added dropwise into sodium cholate solution (1% w/v in H_2_0, 4 mL) while vortexing and then probe sonicated on ice (3 × 10 s). The resulting emulsion was dispersed in 20 ml of 0.3% w/w sodium cholate solution held at 4°C. This fabrication process was repeated three times to produce a total of 200 mg of polymer and 8 mg of adapalene added to the same 20 mL of sodium cholate solution. Following 3 h of solvent evaporation, solutions were passed through a 0.22 μm sterile bottle top filter to remove drug aggregates. Particles were further washed and concentrated with Amicon Ultra-15 Centrifugal filters (MWCO 100k).

### Nanoparticle Characterization

Hydrodynamic diameter, polydispersity index, and zeta potential was measured with a Nanobrook 90 Plus Zeta instrument (Brookhaven) on lyophilized samples resuspended in 1mM KCl at a concentration of 1 mg/mL. Nanoparticle aliquots were lyophilized and weighed to determine percent drug loading (DL). The control curves were used to determine the amount of adapalene per 1 mg/ml of lyophilized nanoparticles to measure drug DL (Villiers et al., 2008); DL = A⁢d⁢a⁢p⁢(m⁢g)1⁢(m⁢g)⁢l⁢y⁢o⁢p⁢h⁢i⁢l⁢z⁢e⁢d⁢n⁢a⁢n⁢o⁢p⁢a⁢r⁢t⁢i⁢c⁢l⁢e⁢X⁢ 100. Transmission Electron Microscopy (TEM) was performed using a JEOL 1200 microscope at an accelerating voltage of 80kV and a magnification of 120k. Specimens were prepared by dipping a mesh copper grid into a 0.1 mg/ml suspension of NPs. The grid was then dried prior to imaging.

### Measuring Retinoid Activation With Lac-z Reporter Mice

Mice, expressing LacZ at the retinoic acid response element (RARE) sites, were obtained from The Jackson Laboratory (Stock# 008477; Tg(RARE-Hspa1b/lacZ)12Jrt/J). Mice were injected with 0, 1.5, 3, or 6 mg/kg via the lateral tail vein and euthanized by transcardial perfusion 24 h later. The brain was removed, fixed in 4% paraformaldehyde PFA in PBS for 48 h, and then cryoprotected in 30% sucrose for 48 h at 4°C. Sections were collected at 30 μm and applied to positively charged glass slides. Slides were washed in staining buffer (1M Magnesium Chloride and 10% Sodium Deoxycholate in H_2_O), followed by an overnight incubation in staining solution (1 mg/mL 5-bromo-4-chloro-3-indolyl-β-D-galactopyranoside (X-gal), 50 mM Potassium Ferricyanide, and 50 mM Potassium Ferrocyanide in staining buffer) at 37°C. The slides were then washed in 1X PBS and coverslipped.

### Animals

All experimental procedures involving animals were approved by the Institutional Animal Care and Use Committee at Barrow Neurological Institute. Female wild type C57BL/6J (Stock number: 000664) (*n* = 40) and B6.Cg-Tg(SOD1^∗^G93A)1Gur/J (Stock number: 004435) transgenic mice (*n* = 52) were obtained from The Jackson Laboratory (Gurney et al., 1994; Wooley et al., 2005). Mice were housed within temperature and humidity-controlled rooms, on 12-h-light/12-h-dark light cycle, and with food and water available *ad libitum*. Mice maintained for lifespan analysis reached endpoint when they could not right themselves within 15 s of being placed on their back. Disease onset was defined as the age at which mice reached their peak weight. Further disease progression was defined as the number of days from disease onset to the day the mice reached their endpoint.

### Treatment Groups

Wild-type and SOD1^*G*93*A*^ transgenic mice were treated with either 3 mg/kg (adapalene) of Adap-NPs or a matched concentration of drug-empty nanoparticles (Ctl-NPs) suspended in sterile saline. Mice were injected with a total volume of 150–200 μl depending on weight. Treatment began when mice reached day 61 days-of-age and consisted of three-times weekly i.v. injection of nanoparticles via the lateral tail vein. For survival analysis, SOD1^*G*93*A*^ mice were randomly divided into Adap-NP treated (*n* = 23) and Ctl-NP treated (*n* = 14) groups. In addition, SOD1^*G*93*A*^ mice were also euthanized at predetermined time points of 104 days (early disease) (Adap-NP *n* = 4; Ctl-NP *n* = 3) and 143 days of age (mid-disease) (Adap-NP *n* = 4; Ctl-NP *n* = 4). No mice were censored from the reported analyses.

### Behavioral Tests

Testing was performed by a researcher blinded to mouse genotype and treatment group. *Rotarod testing:* The week prior to initiating training with the rotarod, mice were individually handled for 5 days for 1 min each day. Mice were trained on the rotarod with an accelerating paradigm, increasing the speed from 4 to 40 rpm over 300 s (Bennett et al., 2014). During training, mice were given 3 trials per day for 3 consecutive days. Nanoparticle treatment began the week after training, during which mice were tested with the rotarod once weekly with three trials per session. The best time of the three trials was used as the measure of motor function (Hayworth and Gonzalez-Lima, 2009; Bennett et al., 2014). *Open field test:* During the 9th week of treatment, mice were allowed to roam freely in a transparent enclosure (37 × 37 cm) for 8 min. The number of rearing episodes (when a mouse stood upright on hind paws in an upright position) was recorded. *Wire hanging test:* During the 10th week of treatment, mice were placed on a metal wire. The number of seconds it took the mouse to fall was recorded.

### Euthanasia and Perfusions

Mice were anesthetized via an intraperitoneal injection of a ketamine (100 mg/kg) and xylazine (10 mg/kg). Transcardial perfusions with cold 1x PBS were performed using an FH100 Peristaltic Pump. The left hemisphere of the brain and spinal cord were removed and frozen on dry ice before being stored at −80°C. The right brain hemisphere, liver, and hindlimbs were removed and fixed in 4% PFA solution in PBS at 4°C. After 48 h in PFA, tissue samples were incubated in 30% sucrose at 4°C for another 48 h. After fixation, the gastrocnemius muscle was dissected for subsequent tissue sectioning. Muscle was cryosectioned at 16 μm and collected on positively charged slides.

### ChAT and GFAP Immunofluorescence

Spinal cord sections from the L3–L5 regions were washed twice for 5 min in 1X TBS with 0.3% Triton X-100 and incubated in SuperBlock (ScyTek) with 0.3% Triton X-100 for 1 h at room temperature. Sections were the incubated with Choline acetyltransferase (CHAT) primary antibody (1:500) (Proteintech # 20747-1-AP) or glial fibrillary acidic protein (1:500) (GFAP) (Dako Cat# Z0334) diluted in 1% Bovine Serum Albumin (BSA) and 0.3% Triton X-100 in 1X TBS (1:500) overnight at 4°C. Following two 5-min washes with 0.3% Triton X-100 in 1X TBS, slides were incubated with Alexa Fluor^TM^ 488 goat anti-rabbit IgG H&L diluted in 1% BSA and 0.3% Triton X-100 in 1X TBS (1:500) for 2 h at room temperature. Slides were rinsed in 1X TBS and mounted with Fluoroshield^TM^ with DAPI. For quantification of spinal motor neurons, confocal images of CHAT labeled lumbar ventral horn were captured and quantification of motor neurons was done by counting the polygon-shaped neurons with cell body diameter of at least 20 μm and a distinct nucleus (Mancuso et al., 2014; Riancho, 2016). At least five sections per mouse were quantified from three different mice per group. GFAP positive cells were quantified by counting GFAP positive cells bodies in the ventral horn that were positive for DAPI from 40x confocal images with an *n* = 3 per group.

### Neuromuscular Junction Innervation

Immunofluorescence was used to quantify the number of innervated and denervated neuromuscular junctions in gastrocnemius muscle sections by a blinded scorer. Neuromuscular junctions were categorized as denervated if there was less than 50% colocalization between α-bungarotoxin and neurofilament. Percent denervated was calculated by dividing the number of denervated NMJs by total number of all NMJs analyzed, multiplied by 100. For IF staining muscle slices were incubated in 1% calcium chloride and trypsin in 1X TBS for 40 min at 37°C for antigen retrieval. Sections were next washed twice for 5 min in 1X TBS with 0.3% Triton X-100, then incubated in Super Block (ScyTek) with 0.5% Triton X-100 for 1 h at room temperature. Slides were then incubated with neurofilament light polyclonal antibody (Millipore Cat# AB9568) diluted in 1% Bovine Serum Albumin (BSA) and 0.3% Triton X-100 in 1X TBS (1:500) overnight at 4°C. Following two 5-min washes with 0.3% Triton X-100 in 1X TBS, slides were incubated with Alexa Fluor^TM^ 488 goat anti-rabbit IgG (1:500) and α-bungarotoxin (1:500) (Thermofisher Scientific Cat#T1175) diluted in 1% BSA and 0.3% Triton X-100 in 1X TBS for 2 h at room temperature. Slides were rinsed in 1X TBS and mounted with Fluoroshield^TM^ with DAPI. An Observer Z1 confocal microscope (Zeiss) with a 40X water objective and Zen 2009 software were used to obtain high-resolution images of neuromuscular junctions.

### Muscle H&E Staining

Slides were submerged in Hematoxalin for 3 min, rinsed in DI water, and submerged in tap water for 5 min. To de-stain, the slides were rapidly dipped 10 times into acid ethanol (70% ethanol and 5 drops acetic acid in DI water), followed by two washes in tap water and a 30 s incubation in eosin. The slides were then incubated in 95% ethanol for 15 min, 100% ethanol for 15 min, and Histochoice^®^ Clearing Agent for 45 min. Organo/Limonene Mount^TM^ was applied and slides were cover slipped. A light microscope and digital camera (Nikon DS-Fi1) was used to image the tissue. Muscle fiber diameter was quantified from longitudinal sections using the NIH ImageJ software.

### Iba-1 IHC

Spinal cords were cryosectioned at 16 μm and collected on microscope slides. The sections were washed with TBS (3 × 10 min) and submerged in 64% methanol/30% H_2_O_2_ diluted with DI H_2_O for 30 min. Slides were then washed with TBS and 0.3% Triton X-100 (3 × 10 min) and incubated in Super Block and 0.3% Triton X-100 for 1 h. Sections were then incubated in rabbit anti-Iba1 primary antibody (Wako #019-19741) was diluted (1:1000) in TBS, 0.3% Triton X-100 and 1% BSA and applied to slides for overnight incubation at 4°C. Slides were washed with TBS (3 × 10 min), incubated with the biotinylated anti-rabbit secondary antibody diluted (1:1000) in Super Block for 1 h, and washed with tris-buffered saline (TBS) (2 × 10 min). The slides were then incubated with the ABC reagent for 1 h, washed with phosphate buffered saline (PBS) (2 × 10 min), rinsed with DI H_2_O, developed with the Vector NovaRED Substrate Kit for exactly 5 min, and washed with DI H_2_O (2 × 5 min). Slides were cleared as follows: 95% ethanol (2 × 10 s), next 95% ethanol (2 × 10 s), 100% ethanol (2 × 10 s), next 100% ethanol (2 × 10 s), and xylene (1 × 10 s). Excess liquid was removed and slides were cover slipped with Limonene mounting media. Microglia were quantified from 20x IHC images by counting Iba1 positive cells in the ventral horn of slices taken L3–L5. An *n* = 3 per group was used with 2–4 slices per mouse being averaged.

### Statistics

Survival data was compared and analyzed using a Kaplan–Meier curve followed by the Gehan–Breslow–Wilcoxon test. Data were analyzed using Student’s *t*-test or two-way ANOVA followed by Tukey’s *post hoc* test using GraphPad 10 software. Normality was tested using Shapiro–Wilk test (S–W), which is appropriate for small samples. Results are presented as mean ± the SEM.

## Results

### Systemically Administered Adap-NPs Induce Retinoid Signaling Within the CNS

**FIGURE 1:**
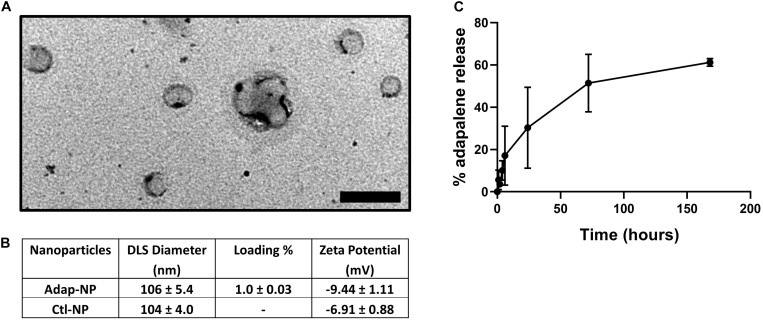
Characterization of adapalene loaded nanoparticles (Adap-NPs). **(A)** Transmission electron microscopy of Adap-NPs. Scale bar = 200 nm. **(B)** Biophysical properties of Adap-NPs. **(C)** Release profile of Adap-NPs demonstrates controlled release of adapalene over 168 h (7 days).

Adapalene-loaded nanoparticles composed of poly(lactic acid)-poly(ethylene glycol) were produced by single emulsion as previously described (Medina et al., 2019). Adap-NPs were spherical in morphology when imaged with Transmission Electron Microscopy (TEM) (Figure 1A). Drug-empty control nanoparticles (Ctl-NPs) and adapalene-loaded nanoparticles (Adap-NPs) possessed mean hydrodynamic diameters of 104 ± 4.0 nm and 106 ± 5.4 nm and surface charges of −6.9 ± 0.9 and −9.4 ± 1.1, respectively. Adapalene was effectively encapsulated within the nanoparticles with an average loading of 1.0 ± 0.03% w/w (*n* = 7 independent batches) (Figure 1B). This reflected an encapsulation efficiency of 39.4 ± 1.25%. The release profile of Adap-NPs was determined *in vitro* in PBS at 37°C (Figure 1C). Approximately 30% of adapalene was released within 24 h. A second, sustained phase of adapalene release was observed over 7 days.

**FIGURE 2:**
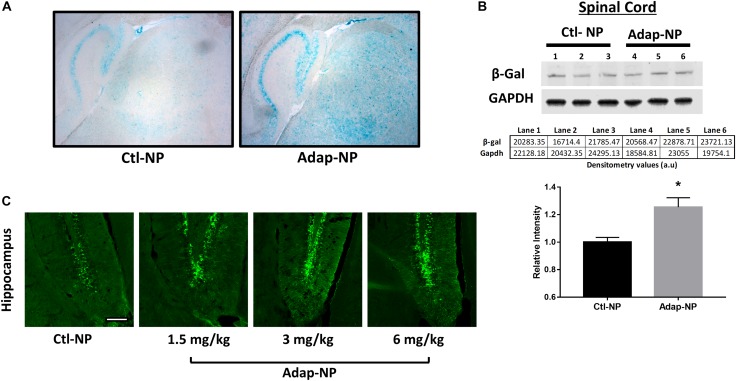
Confirmation of biological activity of Adap-NPs. **(A)** Retinoid signaling reporter mice were sacrificed 24 h after 3 mg/kg of adapalene NP administration and stained with X-gal to image the lacZ reporter gene expression in the brain (blue), and demonstrated the biological activity beta gal induced by peripheral Adap-NP administration (*n* = 3/group). **(B)** Western blot and quantification of β-galactosidase from lysates of spinal cord in reporter mice demonstrating increased retinoid signaling 4 h after 3 mg/kg Adap-NP administration. *p < 0.05 Student’s *t*-test (*n* = 3/group). **(C)** Immunofluorescence for β-galactosidase in the dentate gyrus of the hippocampus following administration of Adap-NPs at 1.5, 3, or 6 mg/kg i.v. injections. Tissue was collected 24 h after i.v. administration. Scale bar = 200 μm.

To assess the biological activity of Adap-NPs and develop dosing paradigms *in vivo*, we utilized reporter mice that express the β-galactosidase (lacZ) gene under the control of the retinoic acid response element (RARE) (Rossant et al., 1991). Staining with X-gal to visualize lacZ expression demonstrated that intravenous administration of Adap-NPs at 3 mg/kg induced retinoid signaling after 24 h throughout the CNS, including in the cerebellum, cortex, and striatum (Figure 2A). Western blot analysis of Adap-NPs treated mice also demonstrated increased β-galactosidase expression in the spinal cord of Adap-NP treated mice compared to controls (Figure 2B) (*p* = 0.02). All doses activated retinoid signaling within the CNS. Doses of 3 or 6 mg/kg activated retinoid signaling to a higher extent than 1.5 mg/kg, although there was no difference between 3 and 6 mg/kg 24 h post administration (Figure 2C). For therapeutic studies, 3 mg/kg was chosen as the minimally effective biological dose.

### Systemic Administration of Adap-NPs Increases Lifespan in SOD1^*G*93*A*^ Mice

**FIGURE 3:**
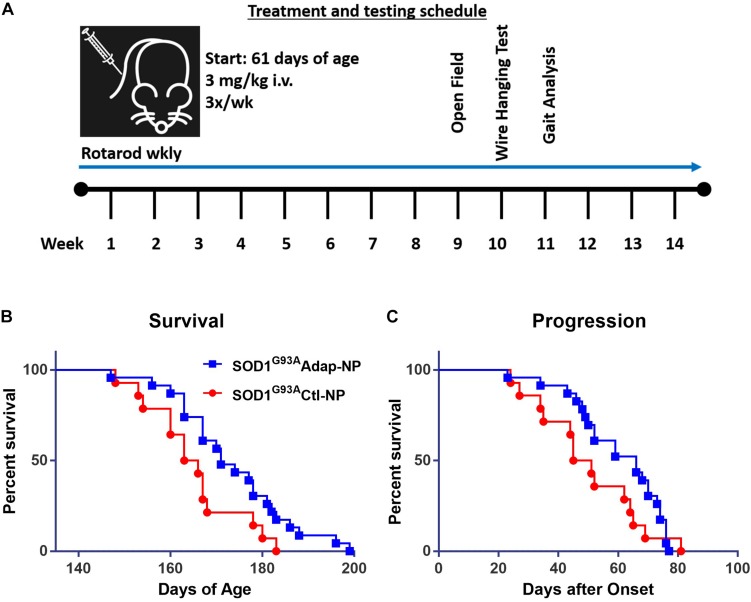
Treatment with Adap-NPs significantly increases lifespan by slowing progression in SOD1^*G*93*A*^ mice. **(A)** Schematic of nanoparticle administration and behavioral assay schedule. **(B)** Kaplan–Meier analysis of survival (Blue line = Adap-NPs; Red line = Ctl-NPs). Average lifespan for SOD1^*G*93*A*^ Ctl-NP mice = 164.5 days compared to SOD1^*G*93*A*^ Adap-NP = 171 days (Gehan–Breslow–Wilcoxon test, *p* = 0.03). Max Life span for SOD1^*G*93*A*^ Ctl-NP = 183 days compared to SOD1^*G*93*A*^ Adap-NP = 199 days. **(C)** Survival plot showing significant delay in disease progression rate as calculated by time from disease onset (age at peak weight) to endpoint (Gehan–Breslow–Wilcoxon test, *p* = 0.04). *n* = Ctl-NP = 13; Adap-NP = 23 mouse per group.

The effect of retinoid modulation on disease progression was evaluated in the SOD1^*G*93*A*^ transgenic mouse model of ALS (Gurney et al., 1994; Wooley et al., 2005). Wild type C57BL/6J mice were used as disease controls. All mice began nanoparticle treatment at 61 days of age. Treatment consisted of lateral tail vein injections three times per week at a dosage of 3 mg/kg of adapalene (Figure 3A). Nanoparticle treatment was well tolerated by mice. No signs of toxicity associated with treatment were observed at any point during the study. Median survival increased significantly from 164 to 171 days for Adap-NP treated mice compared to Ctl-NP mice (Figure 3B; *p* = 0.03, Gehan–Breslow–Wilcoxon test). In addition, the maximum lifespan of transgenic mice was extended from 183 to 199 days for Adap-NP treated mice compared to Ctl-NP mice. The increase in lifespan was not associated with delayed onset of disease, which was defined as age to peak weight (Supplementary Figure 1). However, Adap-NP treatment was associated with a significant delay in progression of disease, which is defined as the number of days between initial onset and endpoint (Figure 3C). Adap-NP treated SOD1^*G*93*A*^ mice survived an average of 69 days after onset compared to 50 days for Ctl-NP treated mice (*p* = 0.04, Gehan–Breslow–Wilcoxon test).

### Systemic Administration of Adap-NPs Reduces Motor Impairment in SOD1^*G*93*A*^ Transgenic Mice

**FIGURE 4:**
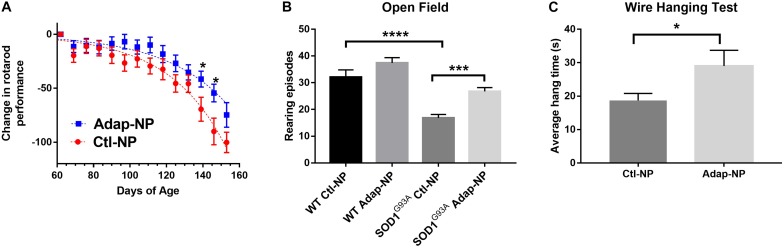
Treatment of SOD1^*G*93*A*^ with Adap-NPs significantly reduces motor impairments. **(A)** Normalized plot demonstrating change in performance on the accelerating rotarod task (4–40 rpm for 300 s) from baseline (average time from week 1) (*p < 0.05; multiple *t*-test). **(B)** Open field test performed at 9 weeks of treatment (119 days of age). Number of rearing episodes in an 8-min open field task (***p < 0.001, ****p < 0.0001; Two-way ANOVA with Tukey’s *post hoc* test). **(C)** Wire hanging test given at 10 weeks of treatment (126 days of age). Average wire hanging times of three trials (*p < 0.05; Student’s *t*-test) *n* = Ctl-NP = 17; Adap-NP = 26.

To analyze the effect of Adap-NP treatment on motor performance, mice were subjected to a battery of behavioral assays. For rotarod testing, each mouse received three trials per week, and the best time out of that week’s trial was recorded. No significant differences were observed in motor performance for treated versus untreated mice in early study stages. With time, transgenic mice treated with Adap-NPs declined in rotarod performance at a slower rate compared to transgenic mice treated with control nanoparticles (Figure 4A). Significant differences in motor performance were observed at 12 weeks (71.4 ± 6.0 s compared to 60.1 ± 5.4 s for Adap-NP versus Ctl-NP treated mice) and at 13 weeks (65.2 ± 5.5 s compared to 43.4 ± 6.8 for Adap-NP versus Ctl-NP treated mice). These differences represented significant declines when measuring performance relative to baseline (a decline of 41.5 ± 7.3% to 54.4 ± 8.4% for Adap-NP treated mice versus 69.4 ± 11% to 90.0 ± 12.3% for Ctl-NP treated mice between 12 and 13 weeks; *p* = 0.02 and *p* = 0.03 for comparing Adap-NP and Ctl-NP declines at weeks 12 and 13 respectively). Adap-NP treated transgenic mice tended to perform better than Ctl-NP treated mice at 14 weeks, although this difference was not statistically significant (*p* = 0.08) (Figure 4A).

Total activity of mice was analyzed in the open field test 9 weeks after initiation of treatment. We quantified the number of rearing episodes during an 8-min trial to assess overall muscle strength and balance. No differences in rearing were observed for Adap-NP versus Ctl-NP treated wild type mice. However, transgenic mice treated with Adap-NPs showed significantly greater rearing compared to Ctl-NP treated mice (an average of 28 ± 1.4 versus 17 ± 1.3 rearing episodes, *p* = 0.0006, Figure 4B). As another measure of muscle strength, mice were also tested using the wire hanging test after 10 weeks of treatment. Mice were allowed to suspend from a thin wire, using both forelimbs and hind limbs to support their body weight. Time to fall was measured over three trials. Transgenic mice treated with Adap-NPs were able to hang significantly longer than transgenic mice treated with Ctl-NP (an average time of 29 ± 3.8 versus 19 ± 2.6 s, *p* = 0.03, Figure 4C).

### Treatment With Adap-NPs Is Neuroprotective and Preserves Motor Units in SOD1^*G*93*A*^ Transgenic Mice

**FIGURE 5:**
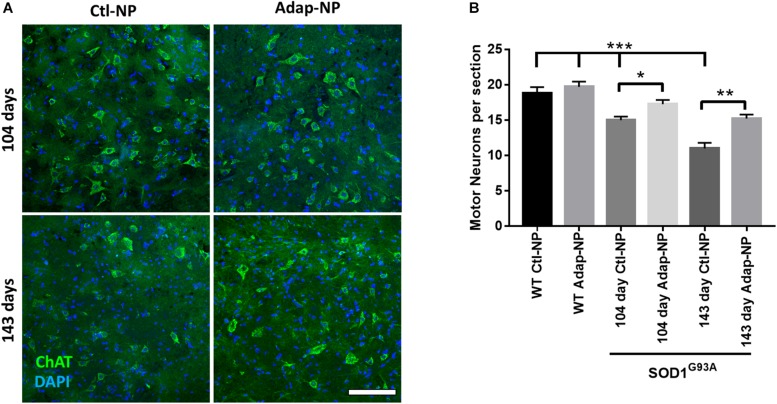
Treatment with Adap-NPs significantly reduces neurodegeneration. **(A)** IF for ChAT (green) to identify motor neurons in the lumbar spinal cord at 104 (top row) and 143 days of age (bottom row). **(B)** Quantification of motor neurons in lumbar spinal cord shows progressive loss of motor neurons in spinal cord is reduced with Adap-NP treatment (*p < 0.05, **p < 0.01, ***p < 0.001; two-way ANOVA with Tukey’s *post hoc* test) *n* = 12–20 sections per group. Scale bar = 100 μm.

We next investigated how treatment impacts maintenance of spinal motor neurons, neuromuscular junctions, and muscle volume at early and late time points of disease progression. Cohorts from each group were euthanized at 104 days of age or 143 days of age. Motor neurons in the ventral horn of the lumbar spinal cord were visualized using choline acetyltransferase (ChAT) immunofluorescence. As expected, the number of motor neurons progressively decreased over time in transgenic mice compared to wild type mice (Figures 5A,B). At 104 days of age, transgenic mice treated with Adap-NPs possessed a greater number of motor neurons in the spinal cord than transgenic mice treated with Ctl-NPs: an average of 17.7 ± 0.62 motor neurons per section compared to 15.0 ± 0.5 motor neurons per section for Adap-NP vs. Ctl-NP treated mice, respectively (*p* = 0.049). Similarly, at 143 days of age transgenic mice treated with Adap-NPs also possessed a greater number of motor neurons in the spinal cord than transgenic mice treated with Ctl-NPs: an average of 15.2 ± 0.63 motor neurons per section compared to 11.0 ± 0.87 motor neurons per section for Adap-NP vs. Ctl-NP treated mice, respectively (*p* = 0.002). Thus, treatment with Adap-NPs significantly reduced loss of motor neurons in the spinal cord at both 104 and 143 days of age.

**FIGURE 6:**
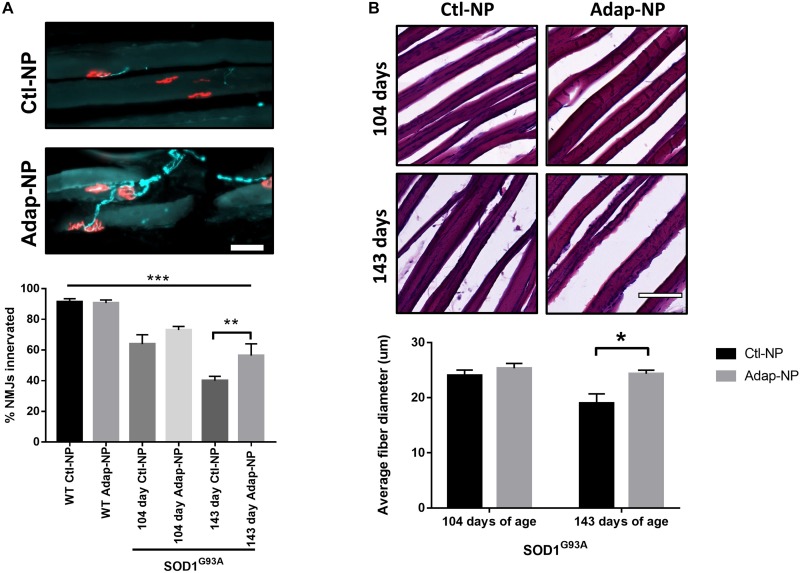
Treatment with Adap-NPs reduces the degeneration of motor units in the SOD1^*G*93*A*^ mice. **(A)** Top: IF of neuromuscular junctions (nmj) at 143 days of age using neurofilament (green) and α-bungarotoxin (red) Bottom: Quantification of nmj innervation demonstrates progressive loss of innervation in transgenic mice which is reduced with Adap-NP. (**p < 0.01, ***p < 0.001; two-way ANOVA with Tukey’s *post hoc* test). **(B)** Top: H&E stained muscle fibers from 104 days old (top row) and 143 days old (bottom row) transgenic mice. Bottom: Quantification shows that Adap-NPs decrease muscle fiber atrophy compared to control. *p < 0.05, Student’s *t*-test. Scale bar = 100 μm. *n* = 3/group.

Treatment effects on neuromuscular junction integrity were measured in the gastrocnemius muscle. Loss of co-localization of neurofilament and bungarotoxin was used to assess denervation. As expected, neuromuscular junction innervation significantly decreased between 104 and 143 days of age in transgenic mice compared to wild type mice (Figure 6A). Mice treated with Adap-NPs demonstrated a trend of reduced denervation at 104 days of age, with 72 ± 2.6 versus 64 ± 6.1% of neuromuscular junctions measured intact for Adap-NP treated versus Ctl-NP treated transgenic mice (*p* = 0.08) (Figure 6A). Significant treatment effects were observed at 143 days of age (56 ± 7.8 compared to 40 ± 2.3% intact neuromuscular junctions for Adap-NP versus Ctl-NP treated transgenic mice, respectively, *p* = 0.007, Figure 6A).

Muscle fiber diameter from the gastrocnemius muscle was measured to determine the effects of Adap-NP treatment on muscle loss. As expected, muscle fiber diameter significantly decreased between 104 and 143 days of age in transgenic mice (Figure 6B). At day 104, there were no significant differences between treated versus untreated mice in either transgenic mice or wild type controls. However, by 143 days of age SOD1^*G*93*A*^ mice muscle fiber diameter was significantly reduced compared to WT (Supplementary Figure 2, *p* = 0.005). Importantly, at day 143, the average muscle fiber diameter was significantly larger in transgenic mice that were treated with Adap-NPs compared to transgenic mice that were treated with Ctl-NPs (an average diameter of 24 ± 0.67 μm versus 19 ± 1.3 μm, respectively, *p* = 0.04, Figure 6B).

### Treatment With Adap-NPs Reduces Iba1 the Spinal Cords of SOD1^*G*93*A*^ Transgenic Mice

**FIGURE 7:**
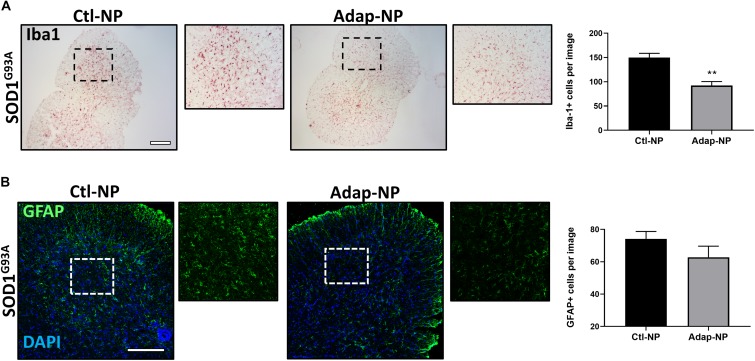
Treatment with Adap-NPs reduces Iba1 in the spinal cords of SOD1^*G*93*A*^ transgenic mice. **(A)** IHC images of microglial marker Iba1 taken from the ventral horn of the spinal cord from 143-day old SOD1^*G*93*A*^ mice. Black box marks where magnified images were acquired (to the right of lower mag images). Quantification reveals significant decrease in Iba1 positive cells after Adap-NP treatment (*p* = 0.008). Scale bar = 500 μm. *n* = 3/group. **(B)** IF images of GFAP (green) taken from the ventral horn of the spinal cord from 143-day old SOD1^*G*93*A*^ mice. White box marks where magnified images were acquired (to the right of lower mag images). Scale bar = 200 μm. Graph represents the quantification of GFAP images.

Progressive increases in astrogliosis and microglial activation over time have been demonstrated in the SOD1^*G*93*A*^ mouse model (Yang et al., 2011). We thus sought to determine the effects of Adap-NP on neuroinflammatory markers within the spinal cords. We found that Iba-1 labeled activated microglia in the spinal cord were significantly reduced by treatment with Adap-NPs (Figure 7A). Quantification revealed a significant reduction of average microglia per image in the ventral horn of the spinal cord in Adap-NP treated mice (92.3 ± 8.03) compared to Ctl-NP treated mice (149.8 ± 8.7) (*p* = 0.008). In addition, Adap-NP treatment tended to reduce GFAP staining in the ventral horn of the spinal cord in SOD1^*G*93*A*^ transgenic mice when compared to mice treated with Ctl-NP, although this change was not significant (Figure 7B).

## Discussion

Several lines of evidence have implicated retinoid signaling in ALS. Dietary deprivation of vitamin A in rats, which leads to loss of retinoid signaling, has been shown to cause phenotypes similar to ALS such as motor impairments, lower motor neuron loss, and inflammation within the spinal cord (Corcoran et al., 2002). Further, several genes that encode proteins of the retinoid signaling pathway are alternatively expressed in post-mortem tissues of ALS patients and animal models of ALS (Jokic et al., 2007; Malaspina and Michael-Titus, 2008). Recent evidence for retinoid signaling as a potential therapeutic target for ALS came from Riancho and colleagues, who demonstrated that chronic administration of a micronized retinoid, bexarotene (Targretin^TM^), can increase lifespan, reduce motor impairments and also be neuroprotective (Riancho et al., 2015). Surprisingly, administration of all-trans RA accelerated ALS symptoms in an ALS mouse model, thus suggesting that indiscriminate activation of RA signaling, versus targeting select receptors, may not be an appropriate ALS treatment strategy (Crochemore et al., 2009).

In this study, we focused on activation of RARβ using a highly specific agonist, adapalene. Adapalene is a third-generation retinoid that has been shown to have high selectivity for RARβ compared to other RARs, and is also more stable and potent than RA (Shroot and Michel, 1997; Michel et al., 1998). Although adapalene is FDA approved for topical treatment of acne and cervical neoplasias, it is poorly water soluble and cannot be systemically administered in biologically relevant quantities, even if delivered at its maximum aqueous solubility from an osmotic minipump. Adapalene experiences a problem that has been a widespread impediment to therapeutic development for ALS and other neurological and neurodegenerative diseases: the inability to deliver drug candidates into the CNS at therapeutically relevant doses. To address this problem, we turned to nanoparticle encapsulation. Encapsulation of small molecules within nanoparticles can increase delivery, reduce toxicities, and enhance CNS activity, even when nanoparticles have not been designed for specific passage across the blood-brain barrier (Cook et al., 2015; Householder et al., 2015, 2018). Importantly, our prior studies demonstrated neuroprotective effects of adapalene in primary spinal motor neuron cultures (Kolarcik and Bowser, 2012) and we hypothesized a nanoparticle formulation would also have low to no toxicity *in vivo*. To that end, our novel formulation did not exhibit signs of toxicity (e.g., weight loss, changes in activity) *in vivo* following both acute administration (Medina et al., 2019) or chronically (over several months) as demonstrated in this study. We previously optimized the design of Adap-NPs and demonstrated that they are capable of robustly activating retinoid signaling throughout the central nervous system following intravenous administration (Medina et al., 2019). Here, we sought to evaluate these nanoparticles in a murine model of ALS, to establish a role of retinoid signaling in ALS pathology and to advance nanoparticle strategies toward clinical application in neurodegeneration.

Chronic administration of Adap-NPs resulted in significant prolongation in the lifespan in SOD1^*G*93*A*^ mice, as evidenced by an increase of 7 days in median survival and a 16-day extension of the maximum lifespan. These results are comparable to prior results in SOD1^*G*93*A*^ mice with the RXR agonist bexarotene, which produced a 10 day increase in median survival (Riancho et al., 2015). Bexarotene did produce a delay in disease onset, however they observed an increase in weight in bexarotene treated mice; in contrast we did not observe weight gains in Adap-NP treated mice, which could account for this difference.

To further understand the observed differences in lifespan and motor function, and to establish a mechanistic foundation for Adap-NP efficacy, we examined histopathological and biochemical markers of adapalene activity. Loss of motor neurons is preceded by neuromuscular junction denervation and has been described in both SOD1^*G*93*A*^ mice and patients (Fischer et al., 2004). Adap-NP treatment significantly reduced motor impairments in SOD1^*G*93*A*^ transgenic mice. We found that reduction in motor impairments corresponded with decreases in markers of neurodegeneration and neuromuscular degeneration. Motor neuron loss was reduced approximately 50% in the Adap-NP treated groups at early- and late-time points in the disease, demonstrating the neuroprotective properties of RARβ activation. As expected, more motor neurons in the lumbar spinal cord of Adap-NP treated mice corresponded with more neuromuscular innervation of the gastrocnemius muscle and larger fiber diameter (Figure 6).

Neuroinflammation is a classic hallmark of neurodegenerative diseases, including ALS (McCombe and Henderson, 2011). In this study, we found that activated microglia, measured by Iba1, were significantly downregulated in SOD1^*G*93*A*^ mice following treatment with Adap-NPs (Figure 7A). This finding is in agreement with previous reports that have demonstrated that RA can decrease inflammation, and that specific agonism of RARβ can reduce neuroinflammation (Goncalves et al., 2015; Riancho et al., 2015; Wang et al., 2015). It is unclear if adapalene is working by protecting neurons and thus reducing initiating events that promote inflammation, or if directly lowering inflammation reduces the rate of neurodegeneration. A previous study using an RARβ agonist reduced glial proliferation after *in vivo* neuronal injury by the transfer of PTEN to glial cells via secreted neuronal exosomes (Goncalves et al., 2015). Further, RA signaling via RARβ can reduce secretion of proinflammatory mediators such as TNF-α and iNOS from activated microglia (Dheen et al., 2005). While there was a downward trend in GFAP positive cells in the spinal cord with Adap-NP treatment, this did not reach significance. This raises the possibility that RARβ may preferentially act on factors that modulate microglial activity compared to astrocytes, possibly providing a clue about the cellular targets of Adap-NPs. A previous report evaluating a therapeutic candidate, AMD3100, in the SOD1^*G*93*A*^ mouse also demonstrated reduction in spinal Iba1 while not detecting changes in GFAP (Rabinovich-Nikitin et al., 2016), thus demonstrating that changes in these two neuroinflammation markers are not always connected. As RARβ can influence the expression of hundreds of genes in the CNS (Niewiadomska-Cimicka et al., 2017), it is possible that the effects we observed from Adap-NP treatment resulted from both direct neuroprotection and reduced neuroinflammation. Future studies will be aimed at determining the cellular targets responsible for therapeutic effects of Adap-NPs.

Ultimately, these studies advance a nanomedicine approach for neuroprotection and establish therapeutic relevance of RARβ signaling in a murine model of ALS. Polymeric nanoparticles have been used in the clinic to improve the delivery of various small hydrophobic drugs for multiple diseases (Anselmo and Mitragotri, 2016). Preclinical studies have also demonstrated the use therapeutic potential of nanoparticle approaches in neurodegenerative diseases such as Alzheimer’s and Parkinson’s disease (Heckman et al., 2013; Esteves et al., 2015; Saraiva et al., 2016). Additionally, a handful of groups have attempted to develop nanoparticle systems as therapeutic approaches for ALS *in vivo* (Bondì et al., 2009; Evans et al., 2014; DeCoteau et al., 2016; Chen et al., 2017). Although no solid nanoparticle formulations have advanced to the clinic for neurodegenerative disorders, the advantages that nanoparticles provide is a strong rationale for further pursing these approaches.

Our lab previously demonstrated that delivery of drugs from polymeric nanoparticles can improve the efficacy of therapeutic candidates while reducing toxicity for the treatment of intracranial tumors via enhancements in bioavailability (Zhou et al., 2013; Cook et al., 2015; Householder et al., 2015, 2018). We have previously studied the fate of intravenously administered nanoparticles and encapsulated payloads in the central nervous system, demonstrating that delivery of hydrophobic payloads to the brain and spinal cord can be achieved even if the nanoparticles have not been designed for BBB passage (Cook et al., 2015; Medina et al., 2017). Particle systems serve to enhance total bioavailability of poorly soluble molecules, and enrichment of nanoparticles along the endothelial cells enables passive diffusion of hydrophobic drugs into the parenchyma (Cook et al., 2015; Medina et al., 2017). We and others have shown that nanoparticles can transfer small hydrophobic payloads through contact with cell membranes (Xu et al., 2009; Medina et al., 2017). Presumably this is the mechanism at work here. Based on our model, we propose that the majority of adapalene that reaches the CNS is being transferred from circulating nanoparticles to endothelial cells of the BBB (rather than direct passage of nanoparticles across the BBB), which enables movement of adapalene into the parenchyma of the CNS to activate retinoid signaling. This approach enables activation of retinoid signaling in the CNS without needing to manipulate the BBB or blood-spinal cord barrier (BSCB) or targeting nanoparticles specifically for transport across the BBB or BSCB. The simplicity of this nanoparticle design is an advantage to facilitating eventual production and testing of our therapeutic formulation in the clinic. One avenue for future work will be the optimization of our system for better encapsulation efficiency. These and other approaches to further optimize formulation may be an important step in translating the system to clinical evaluation.

Taking these results in sum, these studies simultaneously validate retinoid signaling as a therapeutic target and advance nanomedicine as a significant approach in the treatment of ALS. We have shown that activation of retinoid signaling in the brain and spinal cord by the RARβ adapalene is neuroprotective, improving motor function, prolong survival, and protecting cells from progressive neurodegeneration in SOD1^*G*93*A*^ transgenic mice. Given the known biocompatibility of PLGA, including in nanoparticle form (Hrkach et al., 2012), and the early evidence for safety and efficacy described here, further development of this platform could advance Adap-NPs as a novel clinical approach for improved treatment of ALS.

## Data Availability Statement

All datasets generated for this study are included in the article/Supplementary Material.

## Ethics Statement

The animal study was reviewed and approved by IACUC at Barrow Neurological Institute.

## Author Contributions

DM was involved in design, execution and analysis of study, and preparation of manuscript. EC and CT were involved in execution of experiments. RB collaborated and oversaw the execution of the study. RS served as senior PI and oversaw the design, execution, and final manuscript preparation.

## Conflict of Interest

We wish to draw the attention of the Editor to the following facts that may be considered as potential conflicts of interest. DM, RB, and RS have recently founded a company (NP Therapeutics, Inc., Phoenix, AZ, United States) whose mission is focused on the development of nanoparticle technologies for the treatment of central nervous system disease. The company does not generate any revenue, does not financially support any scientific endeavors, and is not actively involved in scientific experiments at this time. All experiments described in this manuscript were performed at Barrow Neurological Institute prior to the formation of NP Therapeutics and therefore did not involve the company. The remaining authors declare that the research was conducted in the absence of any commercial or financial relationships that could be construed as a potential conflict of interest.
