# Supplementary material for: Intravenously Administered, Retinoid Activating Nanoparticles Increase Lifespan and Reduce Neurodegeneration in the SOD1G93A Mouse Model of ALS
Source: Front Bioeng Biotechnol. 2020 Mar 27;8:224. doi: 10.3389/fbioe.2020.00224 (PMC7118553; doi:10.3389/fbioe.2020.00224)
Supplement: Supplementary file 1 [file Table_1.DOCX]

**Supplementary Information:**


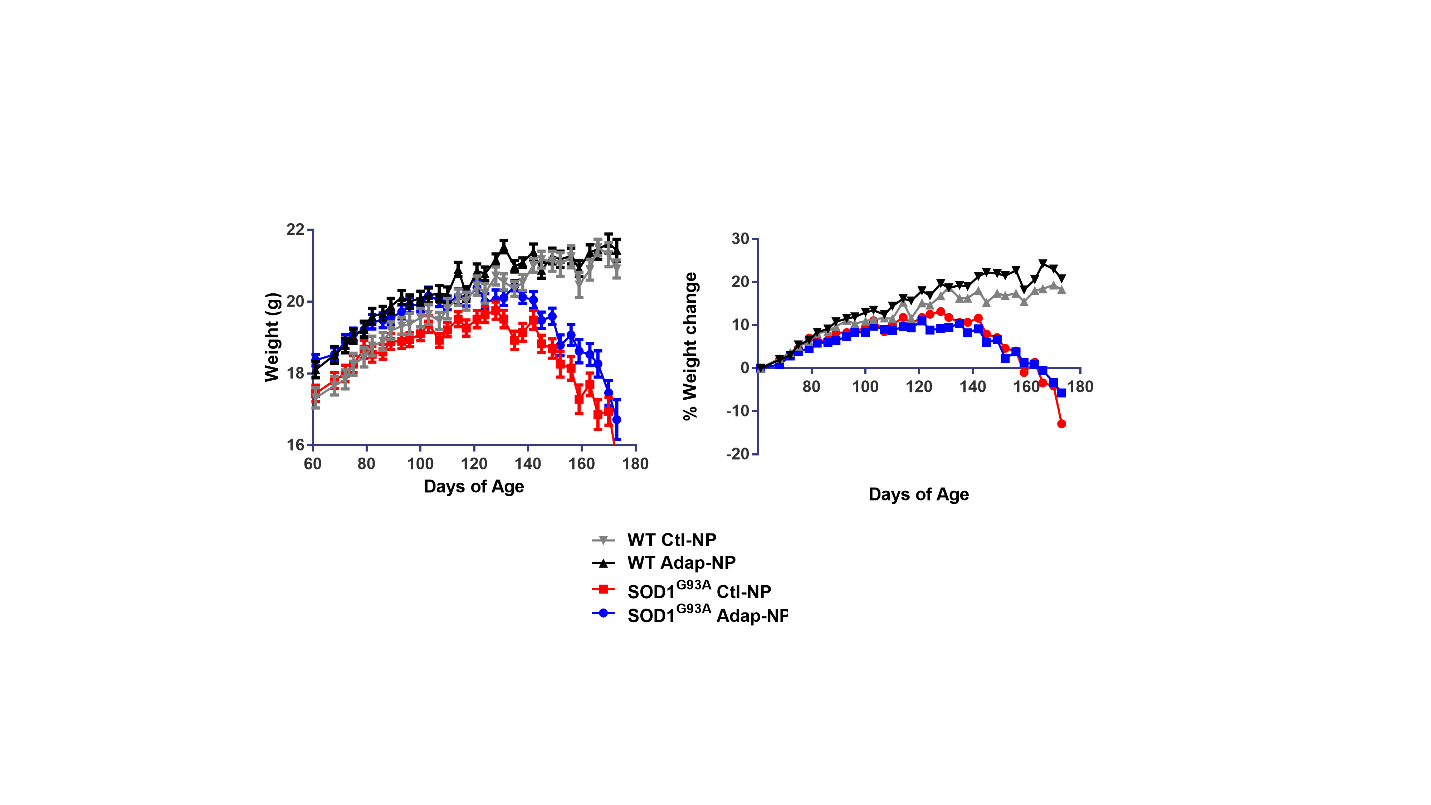


**Supplementary Figure 1. Animal weights by treatment** A) Raw mouse weights beginning at day 60. B) No significant differences in % weight change was observed between SOD1^G93A^ mice on Ctl-NPs or Adap-NPs.
