# Supplementary material for: Intravenously Administered, Retinoid Activating Nanoparticles Increase Lifespan and Reduce Neurodegeneration in the SOD1G93A Mouse Model of ALS
Source: Front Bioeng Biotechnol. 2020 Mar 27;8:224. doi: 10.3389/fbioe.2020.00224 (PMC7118553; doi:10.3389/fbioe.2020.00224)
Supplement: Supplementary file 2 [file Image_2.pdf]

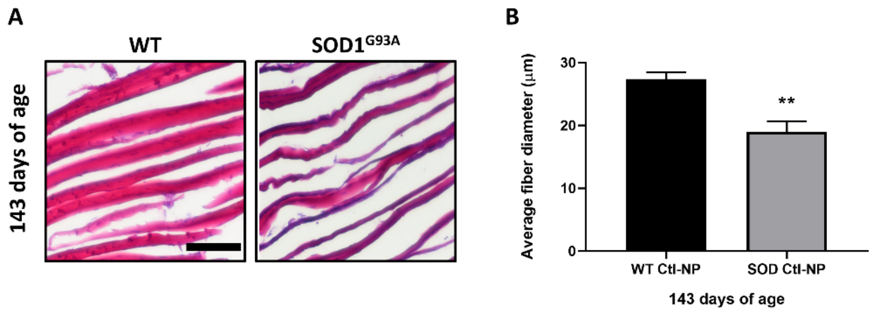

Supplementary Figure 2: Significant loss of muscle fiber size in SOD1^{G93A} mice. A) H&E staining of gastrocnemius muscle at 143 days demonstrate obvious loss of muscle fiber size in SOD1^{G93A} mice compared to WT. B) Quantification shows significant decrease in SOD1^{G93A} mice compared to WT.
